# Evaluation of Risk Factors for Conversion From a COVID-19 Household Contact to a Case in New York City, August 1, 2020, to July 31, 2021

**DOI:** 10.1001/jamanetworkopen.2022.33001

**Published:** 2022-09-14

**Authors:** Katherine Whittemore, Steffen Foerster, Kathleen Blaney, Theodore Long, Neil M. Vora

**Affiliations:** 1 New York City Health and Hospitals, New York; 2 New York City Department of Health and Mental Hygiene, Long Island City

## Abstract

This cross-sectional study examines more than 600 000 household contacts in New York City to characterize the risks of acquiring SARS-CoV-2 infection after household exposure.

## Introduction

SARS-CoV-2 is commonly transmitted in the household, and the risk of converting to a case after a household exposure is generally higher than in other settings, largely reflecting the challenges of quarantine and isolation for persons living in the same dwelling.^1,2^ We analyzed data from more than 600 000 household contacts in New York City (NYC) to characterize the risks of acquiring infection after household exposure.

## Methods

Data for this cross-sectional study were obtained from the NYC Test & Trace Corps (T2), which was created to facilitate widespread COVID-19 testing, contact tracing, and administration of supportive services for those quarantining and isolating in NYC. We used logistic regression to assess risk factors associated with conversion from a household contact to a case. To evaluate the association of vaccination, we included 2 proxy variables: (1) percentage of NYC population in the same age group who were vaccinated for COVID-19 by the month of the contact’s exposure date and (2) a binary variable indicating availability of COVID-19 vaccine in NYC by month of household contact’s exposure. Race and ethnicity were self-reported.

The NYC Health and Hospitals institutional review board determined this study exempt from review because it was part of a public health program evaluation. This study followed the STROBE reporting guideline. Statistical analysis was performed using RStudio version 1.3.1093 (R Project for Statistical Computing) from December 2021 to August 2022. Additional eMethods are detailed in the Supplement.

## Results

During August 1, 2020, to July 31, 2021, of 605 849 household contacts identified, 165 487 (27.3%) were women, 133 724 (22.1%) were men, 56 665 (9.4%) were Black, 121 233 (20.0%) were Hispanic or Latino, 72 976 (12.0%) were White, and 145 899 (24.1%) converted to a case within 2 to 10 days after the first exposure date to the index case while infectious (Table 1).

**Table 1.** Characteristics of COVID-19 Household Contacts in New York City from August 1, 2020, to July 31, 2021

Characteristic	No. (%)	
	Household contacts who did not convert to a case	Household contacts who converted to a case
Overall	459 950	145 899
Borough		
Bronx	92 011 (20.0)	29 918 (20.5)
Brooklyn	132 475 (28.8)	40 101 (27.5)
Manhattan	53 166 (11.6)	16 521 (11.3)
Queens	139 505 (30.3)	45 645 (31.3)
Staten Island	39 174 (8.5)	13 263 (9.1)
Unknown	3619 (0.8)	451 (0.3)
Gender identity		
Female or woman/girl	106 109 (23.1)	59 378 (40.7)
Male or man/boy	87 215 (19.0)	46 509 (31.9)
Transgender/nonbinary or genderqueer/gender identity not listed	444 (0.1)	245 (0.2)
Unknown	266 182 (57.9)	39 767 (27.3)
Race		
Asian, including South Asian	22 799 (5.0)	12 003 (8.2)
Black, including African American or Afro-Caribbean	39 541 (8.6)	17 124 (11.7)
Native Hawaiian or Pacific Islander/Native American or Alaskan Native	1623 (0.4)	829 (0.6)
White	48 220 (10.5)	24 756 (17.0)
Other^a^	33 677 (7.3)	18 345 (12.6)
Unknown	314 090 (68.3)	72 842 (49.9)
Ethnicity		
Hispanic or Latino	78 070 (17.0)	43 163 (29.6)
Not Hispanic or Latino	96 387 (21.0)	46 438 (31.8)
Unknown	285 493 (62.1)	56 298 (38.6)
Age group, y		
0-17	95 991 (20.9)	41 190 (28.2)
18-34	91 145 (19.8)	37 333 (25.6)
35-54	95 119 (20.7)	39 702 (27.2)
55-74	50 200 (10.9)	22 070 (15.1)
≥75	7472 (1.6)	4189 (2.9)
Unknown	120 023 (26.1)	1415 (1.0)
Comorbidities		
Diabetes	10 919 (2.4)	6006 (4.1)
Heart attack	2330 (0.5)	1424 (1.0)
Hypertension	13 818 (3.0)	7053 (4.8)
Immunocompromised	3786 (0.8)	2351 (1.6)
Lung disease	10 388 (2.3)	5662 (3.9)
Overweight	13 185 (2.9)	7318 (5.0)
Smoke or vape	7735 (1.7)	3100 (2.1)
Other	1607 (0.3)	875 (0.6)
No comorbidities reported	396 182 (86.1)	112 110 (76.8)
Health care worker		
Yes	4298 (0.9)	1597 (1.1)
No	28 555 (6.2)	10 225 (7.0)
Unknown	427 097 (92.9)	134 077 (91.9)
Relationship to case		
Aunt or uncle	5873 (1.3)	1155 (0.8)
Child	122 494 (26.6)	44 233 (30.3)
Grandparent	8641 (1.9)	2049 (1.4)
Intimate partner	97 754 (21.3)	39 011 (26.7)
Other	37 398 (8.1)	9285 (6.4)
Parent	107 255 (23.3)	28 707 (19.7)
Roommate	20 454 (4.4)	3565 (2.4)
Sibling	60 081 (13.1)	17 894 (12.3)
Symptomatic index case		
Yes	374 396 (81.4)	135 675 (93.0)
No	75 370 (16.4)	10 224 (7.0)
Unknown	10 184 (2.2)	0

^a^ Includes persons who identified as multiracial or did not identify with any race provided.

The following groups had higher odds of converting to a case: persons with a gender identity of female or woman/girl, persons with a gender identity of transgender/nonbinary or genderqueer/gender identity not listed; Asian and Hispanic or Latino persons; intimate partners, children or siblings of a case; persons exposed to a symptomatic case. Comorbidities associated with higher odds of converting from a household contact to a case included history of diabetes, heart attack, hypertension, immunosuppression, lung disease, and being overweight (Table 2). For each increase in the percentage of persons in the household contact’s age group who were vaccinated against COVID-19, the odds of converting to a case decreased by 1%.

**Table 2.** Results of Multivariable Logistic Regression Model for Converting From a Household Contact to a Case in New York City, August 1, 2020, to July 31, 2021 (N = 452 676)

Risk factor	Adjusted OR (95% CI)
Borough	
Bronx	1.00 (0.97-1.03)
Brooklyn	0.99 (0.97-1.01)
Manhattan	1 [Reference]
Queens	1.03 (1.01-1.05)
Staten Island	1.05 (1.02-1.09)
Unknown	2.37 (1.62-3.50)
Gender	
Female or woman/girl	1.10 (1.08-1.12)
Male or man/boy	1 [Reference]
Transgender or nonbinary or genderqueer	1.22 (1.04-1.43)
Unknown	0.56 (0.55-0.57)
Race	
Asian, including South Asian	1.02 (0.99-1.05)
Black, including African American or Afro-Caribbean	0.86 (0.84-0.88)
Native Hawaiian or Pacific Islander/Native American or Alaskan Native	0.93 (0.85-1.02)
White	1 [Reference]
Other^a^	1.01 (0.98-1.04)
Unknown	1.04 (1.02-1.07)
Ethnicity	
Hispanic or Latino	1.07 (1.04-1.09)
Not Hispanic or Latino	1 [Reference]
Unknown	1.05 (1.02-1.08)
Age (per 10 y)	1.01 (1.01-1.02)
Comorbidity	
Diabetes	1.15 (1.11-1.19)
Heart attack	1.27 (1.18-1.36)
Hypertension	1.06 (1.02-1.09)
Immunocompromised	1.25 (1.19-1.32)
Lung disease	1.13 (1.10-1.17)
Overweight	1.11 (1.08-1.15)
Smoke or vape	0.83 (0.80-0.87)
Other	1.19 (1.08-1.30)
No comorbidities reported	1 [Reference]
Relationship to index case	
Aunt/uncle	0.89 (0.83-0.96)
Child	1.23 (1.20-1.26)
Grandparent	0.99 (0.93-1.04)
Intimate partner	1.37 (1.35-1.40)
Other	1.06 (1.02-1.09)
Parent	1 [Reference]
Roommate	0.76 (0.73-0.79)
Sibling	1.15 (1.12-1.19)
Symptomatic index case	
Yes	2.71 (2.64-2.77)
No	1 [Reference]
% of NYC population in the same age group who were vaccinated for COVID-19 by the month of the contact’s exposure date	0.99 (0.99-0.99)
Availability of COVID-19 vaccine in NYC by month of household contact’s exposure	0.95 (0.92-0.97)
Month of household contact’s	
Exposure	1.34 (1.32-1.37)
Exposure, squared	0.98 (0.98-0.98)

^a^ Includes persons who identified as multiracial or did not identify with any race provided.

## Discussion

Our investigation included more than 600 000 household contacts in NYC, of which 24.1% converted to a case. Our contact-to-case conversion rate is higher when compared with similar studies, such as a pooled estimate of 21.1% reported in a systematic review of 29 studies of COVID-19 secondary attack rates among a total of 22 214 household contacts.^1^

We found that the percentage of persons in NYC in the household contact’s age group who were vaccinated for COVID-19 by the month of the household contact’s exposure had an inverse relationship with converting to a case. This finding suggests that COVID-19 vaccination was protective against converting from a household contact to a case. Public health agencies should continue to prioritize vaccination campaigns for the public.

Women, persons with comorbidities, and persons exposed to a symptomatic case were more likely to convert from a household contact to a case when compared with men. Our findings emphasize the importance of supportive services such as those provided by T2, such as free hotel rooms for isolation and quarantine, allowing for safe separation from household members.

Our investigation had several limitations. First, not all household contacts identified were tested for COVID-19. Second, data for household contacts who did not convert to a case were less complete than data for contacts who converted to a case. Missing data could potentially be related to lower testing probability and subsequent case ascertainment in exposed persons. Third, medical history data including comorbidities were self-reported.

Our investigation suggests that among household contacts in NYC, certain groups were more likely to convert to a case. Better characterizing transmission dynamics among these groups could lead to improved preventative measures and save lives.

## Notes

Supplement. **eMethods.** Supplemental Methods
