# Supplementary material for: Evaluation of Risk Factors for Conversion From a COVID-19 Household Contact to a Case in New York City, August 1, 2020, to July 31, 2021
Source: JAMA Netw Open. 2022 Sep 14;5(9):e2233001. doi: 10.1001/jamanetworkopen.2022.33001 (PMC9475389; doi:10.1001/jamanetworkopen.2022.33001)
Supplement: Supplement. — eMethods. Supplemental Methods [file jamanetwopen-e2233001-s001.pdf]

Supplemental Online Content

Whittemore K, Foerster S, Blaney K, Long T, Vora NM. Evaluation of risk factors for conversion from a COVID-19 household contact to a case in New York City, August 1, 2020, to July 31, 2021. *JAMA Netw Open.* 2022;5(9):e2233001. doi:10.1001/jamanetworkopen.2022.33001

eMethods. Supplemental Methods

This supplemental material has been provided by the authors to give readers additional information about their work.

© 2022 Whittemore K et al. *JAMA Network Open.*

eMethods. Supplemental Methods

Definitions

A household contact was defined as any person living with someone who was a lab-confirmed or probable COVID-19 case while infectious, excluding persons living in congregate settings. A lab-confirmed case occurred in any person with a positive laboratory result (PCR or antigen for COVID-19, not including at-home tests). A probable case occurred in any person who was symptomatic and had an exposure (being within 6 feet for ≥10 cumulative minutes of a person with COVID-19 while that person was infectious) within the past 14 days with no confirmatory lab testing performed for COVID-19. COVID-19 symptoms were defined as fever, cough, chills, shortness of breath, sore throat, muscle aches, diarrhea, headache, nausea or vomiting, confusion, loss of smell or taste, fatigue, or sinus congestion. A household contact's COVID-19 onset date was the earliest reported symptom onset date or date of specimen collection if asymptomatic. A household contact's first exposure date was the date of onset of the infectious period of the index case in the household, which was estimated 2 days prior to the COVID-19 onset date.

Study population

Our study population included NYC residents who were household contacts of a COVID-19 case (lab-confirmed or probable) during August 1, 2020–July 31, 2021.

IRB exemption

Our investigation was considered part of a public health program evaluation and was therefore determined by the institution to be exempt from IRB review.

STROBE reporting guidelines

Our investigation includes all applicable items on the STROBE Statement checklist for cross-sectional studies.

Data analysis

We considered household contacts to have been infected by a household member if their COVID-19 onset date was 2–10 days after the first exposure date to the infectious index case in the household. If a household contact had multiple exposures, only the earliest exposure was used for analysis. We used R for all analyses.

© 2022 Whittemore K et al. *JAMA Network Open.*
